# Maternal prepregnancy weight and gestational weight gain in association with autism and developmental disorders in offspring

**DOI:** 10.1002/oby.23228

**Published:** 2021-08-04

**Authors:** Susana L. Matias, Michelle Pearl, Kristen Lyall, Lisa A. Croen, Tanja V. E. Kral, Daniele Fallin, Li-Ching Lee, Chyrise B. Bradley, Laura A. Schieve, Gayle C. Windham

**Affiliations:** 1 Department of Nutritional Sciences and Toxicology, University of California, Berkeley, California, USA; 2 Environmental Health Investigation Branch, California Department of Public Health, Richmond, California, USA; 3 AJ Drexel Autism Institute, Drexel University, Philadelphia, Pennsylvania, USA; 4 Division of Research, Kaiser Permanente, Oakland, California, USA; 5 Department of Biobehavioral Health Sciences, University of Pennsylvania, Philadelphia, Pennsylvania, USA; 6 Department of Mental Health, Johns Hopkins Bloomberg School of Public Health, Baltimore, Maryland, USA; 7 Department of Epidemiology, Johns Hopkins Bloomberg School of Public Health, Baltimore, Maryland, USA; 8 Department of Epidemiology, Gillings School of Global Public Health, University of North Carolina, Chapel Hill, North Carolina, USA; 9 National Center on Birth Defects and Developmental Disabilities, Centers for Disease Control and Prevention, Atlanta, Georgia, USA

## Abstract

**Objective:**

Maternal prepregnancy BMI and gestational weight gain (GWG) are examined in relation to autism spectrum disorder (ASD) and other developmental disorders (DD) in offspring in a multisite case-control study.

**Methods:**

Maternal prepregnancy BMI, obtained from medical records or self-report, was categorized as underweight, normal weight, overweight, obesity Class 1, or obesity Class 2/3. GWG was standardized for gestational age (GWG *z* score), and the rate (pounds/week) was categorized per adherence with clinical recommendations. Logistic regression models, adjusting for demographic factors, were used to assess associations with ASD (*n* = 1,159) and DD (*n* = 1,617), versus control children (*n* = 1,633).

**Results:**

Maternal obesity Class 2/3 was associated with ASD (adjusted odds ratio [AOR] = 1.87, 95% CI: 1.40–2.51) and DD (AOR = 1.61, 95% CI: 1.22–2.13). GWG *z* score was not associated with DD (AOR = 1.14, 95% CI: 0.95–1.36), but the GWG *z* score highest tertile was associated with higher odds of ASD, particularly among male children (AOR = 1.47, 95% CI: 1.15–1.88).

**Conclusions:**

Results indicate that maternal prepregnancy severe obesity increases risk of ASD and DD in children and suggest high gestational-age-adjusted GWG is a risk factor for ASD in male children. Because maternal BMI and GWG are routinely measured and potentially modifiable, these findings could inform early interventions for high-risk mother-child dyads.

## INTRODUCTION

Autism spectrum disorder (ASD), a neurodevelopmental condition characterized by impairments in social communication and interaction in the presence of restricted, repetitive behaviors (1), affects about 2% of children in the United States (2,3). Although its etiology is not completely understood, evidence indicates that prenatal risk factors are linked to ASD (4–6). In recent meta-analyses, maternal prepregnancy overweight and obesity were associated with increased risk of ASD in children (7–9). However, only one study separately examined maternal obesity severity (10). Furthermore, in one meta-analysis, maternal prepregnancy overweight and obesity were also associated with other developmental disorders (DD), specifically cognitive/intellectual delay (8). One hypothesis of the underlying mechanism relates to the relationship between excessive maternal weight and increased maternal systemic inflammation, which could affect placental function and consequently neurodevelopment in the fetus (11,12).

Limited studies also document associations between gestational weight gain (GWG), in excess of recommendations (13), and both ASD (14–16) and other adverse neurodevelopmental outcomes in children (17,18). This is particularly concerning, given that 48% of US women gain excessive weight during pregnancy (19).

Using data from the first phase of the Study to Explore Early Development (SEED) (20,21), we previously reported an association between maternal prepregnancy obesity and ASD and between both prepregnancy overweight and obesity and other DD among singleton, term births (22). Small sample sizes prevented us from examining obesity severity levels. Given the increasing obesity prevalence in the United States, particularly its most severe form (i.e., Class 3; from 7.4% in 2005 to 2006 to 11.5% in 2017 to 2018 in adult women) (23), a more detailed investigation of severe obesity is needed. In our previous SEED analysis, we also reported an association between greater total GWG and ASD, but not DD, among singleton, term births (22). Because total GWG is naturally correlated with length of gestation, an approach that isolates GWG from gestational duration is suitable for studying the effect of this factor (24). This may be particularly important for studying ASD and other DD, which have been associated with preterm birth (25,26).

A second phase of SEED, which nearly doubled the number of participants, was conducted to allow for more detailed assessments of some associations of interest. The current analysis uses SEED data from phases 1 and 2 to extend our previous analysis. Specifically, we aimed to examine associations of ASD and DD with (1) maternal prepregnancy BMI status, with a particular focus on level of obesity severity, and (2) GWG using a metric that accounts for length of gestation and prepregnancy BMI.

## METHODS

### Study design and sample

SEED is a multisite case-control study that aims (1) to characterize the autism behavioral phenotype and associated developmental, medical, and behavioral conditions and (2) to investigate genetic and environmental risk factors for ASD (20). In the six SEED sites (located in California, Colorado, Georgia, Maryland, North Carolina, and Pennsylvania), eligible children were 2 to 5 years old at enrollment, born between September 2003 and August 2006 (phase 1; *n* = 3,769) or between January 2008 and December 2011 (phase 2; *n* = 3,347), residing in one of these study areas, and with a caregiver who could communicate in English (all sites) or Spanish (California and Colorado). Identification of potential cases (ASD or DD) relied on data from multiple clinical and education sources (see “Outcome ascertainment”). A general population (POP) control group was randomly sampled from children born in the same years from birth certificate data in each study area. Institutional Review Boards at each site and the Centers for Disease Control and Prevention approved the study, and caregivers of enrolled participants provided informed consent.

### Data collection

SEED data collection included (a) an in-person clinic visit to conduct standardized developmental assessments; (b) a computer-assisted telephone interview with the child’s mother to obtain sociodemographic information, child health, maternal reproductive history, and information about her pregnancy with the child; (c) prenatal care and labor and delivery medical records abstraction (when available); and (d) selected information from birth certificates.

### Outcome ascertainment

Details of SEED ASD and DD classification procedures have been previously published (21). Briefly, although the recruitment process included identifying children previously diagnosed with ASD or other DD, on enrollment into SEED, caregivers of all children, including those initially identified through birth certificate sampling for the POP group, were administered the Social Communication Questionnaire (SCQ) (27) to screen for autism symptoms. Children with an SCQ score below 11 and with no previous ASD diagnosis underwent a general in-person developmental assessment, which included administration of the Mullen Scales of Early Learning (MSEL) (28). Children with (1) a previous ASD diagnosis, (2) an SCQ score ≥11, and/or (3) ASD symptoms noted by a SEED research clinician during the general developmental assessment were administered a full ASD developmental evaluation. In addition to the MSEL, these children were administered the Autism Diagnostic Observation Schedule (ADOS) (29), and their caregivers were administered the Autism Diagnostic Interview-Revised (ADI-R) (30); these instruments are considered gold-standard assessment for ASD research. Final SEED ASD classification was based on an algorithm using the ADOS and ADI-R results, which was developed in keeping with best clinical practice guidelines and relevant literature to maximize both sensitivity (0.86) and specificity (0.74) (21). The ASD group was further divided according to whether or not the child had co-occurring intellectual disability (ID; MSEL standard score ≤70). Children who underwent the ADOS and ADI-R but whose scores did not meet the study ASD case criteria were classified as DD or POP, according to their original sampling source (health/education source or birth certificate sample).

### Exposure variables

Prepregnancy BMI and GWG were the two primary “exposures” for this analysis. Information on maternal height and weight before pregnancy and amount of weight gained/lost during pregnancy was obtained from abstraction of prenatal and labor and delivery medical records as the primary source of data. However, because those data were only available for 34% (GWG) to 42% (BMI) of participants, we secondarily relied on self-reported data from the maternal telephone interview. Among women who had data from both sources, we examined weighted Cohen’s kappa statistics and found near perfect agreement between BMI classification from the medical records versus the maternal interview (*n* = 2,189; K_w_ = 0.89) and a moderate agreement for GWG categories (*n* = 1,735; K_w_ = 0.58). We used an established approach developed for a prepregnancy obesity study to identify and remove outliers (31). Outliers that could not be rectified on manual review were set to missing (<1%).

Prepregnancy BMI was calculated as weight/height^2^ (kilograms/meters squared) and classified as underweight (<18.5 kg/m^2^), normal weight (18.5 to 24.9 kg/m^2^), overweight (25.0 to 29.9 kg/m^2^), or obesity Class 1 (30.0 to 34.9 kg/m^2^), Class 2 (35.0 to 39.9 kg/m^2^), or Class 3 (≥40 kg/m^2^) (32). Because of the small cell size, we combined Class 2 and Class 3 obesity into one category for analysis (labeled Class 2/3).

Because GWG is correlated with duration of pregnancy, we standardized GWG for gestational age (GWG-for-GA) by calculating gestational age-specific *z* scores using the methodology and charts described by Hutcheon et al. (33,34). Gestational age at delivery in completed weeks was obtained from birth certificates. Briefly, *z* scores were calculated by comparing a woman’s weight gain to the gestational-week-specific mean and standard deviation of weight gain in a US population, obtained from BMI-specific GWG-for-GA charts (33,34). GWG-for-GA *z* scores were categorized into tertiles based on maternal-BMI-specific distributions (33rd and 66th percentile values for each of the six usual BMI categories; see Supporting Information Table S1) for analysis.

For clinical relevance, we also created a rate of GWG (pounds/week) during the second and third trimester, excluding the first trimester when little weight gain is assumed to occur. We calculated this variable by dividing total pregnancy weight gain minus 4.4 pounds (the maximum weight gain assumed to occur in the first trimester (13)) by gestational age minus 13 weeks (the length of the first trimester). We then created a variable indicating adherence to clinical guidelines for GWG rate per prepregnancy BMI (13); for underweight, 1 to 1.3 pounds/week; normal weight, 0.8 to 1 pounds/week; overweight, 0.5 to 0.7 pounds/week; and obesity, 0.4 to 0.6 pounds/week (13). Based on the Institute of Medicine (IOM) recommendations (13), women’s rate of GWG was classified as “Inadequate” if it was below the recommended range, “Adequate” if within the range, or “Excessive” if it exceeded the range. Secondarily, we categorized the GWG rate variable into tertiles based on the distribution in the POP group (see Supporting Information Table S1 for BMI-specific cutoff values). Less than 4% of mothers were missing information for the GWG variables.

### Covariates

Potential confounders were identified from the literature (35,36) and other SEED analyses (22,37). Demographic variables including maternal age, race/ethnicity, education, and household income were obtained from the maternal interview. Maternal smoking status during peri-conception and pregnancy was derived from interview and prenatal medical records data; parity was obtained from birth certificates. Hypertension and diabetes were not included as potential confounders because they may be in the causal pathway (36–38). We created direct acyclic graphs to guide selection of covariates for adjusted analyses.

### Statistical analysis

For this analysis, we excluded participants with an incomplete interview (*n* = 1,850), children with incomplete data to assign final case classification (*n* = 449), nonbiological mother respondent (*n* = 32), nonsingleton pregnancies (*n* = 284), and women with missing BMI data (*n* = 91). The analytic sample included 4,409 mother-singleton child dyads (1,159 with ASD, 1,617 with DD, and 1,633 in the POP group).

χ^2^ tests were used to assess associations between the exposure variables and case status. Crude and adjusted odds ratios (AOR) were calculated by logistic regression in separate models for ASD, the subgroups ASD with or without ID, or DD, compared with POP controls, for each of the primary exposures of interest, based on a complete case analysis. For maternal prepregnancy BMI, the normal-weight category was the reference. For GWG-for-GA *z* scores (and rate of GWG), the second tertile was the reference category. For GWG rate, per IOM guidelines, gaining “Adequate” weight was considered the reference. Adjusted models included maternal age, education, race/ethnicity, income, parity, smoking, and study site. For GWG, we further adjusted for source of data (medical records vs. interview) because of moderate agreement between sources. For all models, we examined effect modification by child sex. In addition, for GWG models we tested (a) source of data and (b) high BMI (overweight and obesity) versus normal weight, as potential modifiers. We tested these as interaction terms, one at a time, in the adjusted models, using a *p* < 0.10 for significance for a useful gain in power (39). We conducted a sensitivity analysis excluding from the DD group any children with a prior ASD classification or ASD-like traits but not meeting study case criteria for ASD (*n* = 292) to create a clean “non-ASD DD” group for comparison of results for all exposure metrics.

## RESULTS

Participants included in this analytic sample were older (*p* < 0.0001), less likely to be Hispanic (*p* < 0.0001), and had higher maternal education levels (*p* < 0.0001) than those not included (data not shown). There were also more term births (*p* < 0.0001) and female children (*p* = 0.0005) in the analytic sample (data not shown). Children with ASD or DD were more likely to be males and have been born preterm than children in the POP group. Mothers of children with ASD or DD were more likely to be non-Hispanic Black or Hispanic, have lower education and household incomes, and report perinatal smoking and hypertension, compared with POP mothers (Table 1).

Table 2 describes the maternal exposure weight variables by offspring case status. The average maternal prepregnancy BMI was higher for ASD and DD cases than for controls. Mothers of children with ASD or DD were less likely to have a normal prepregnancy BMI and more likely to have a prepregnancy BMI in the obesity Class 2/3 category, compared with mothers of POP children (Table 2). GWG-for-GA *z* scores were higher among mothers of children with ASD, whereas GWG rates (pounds/week) were lower in mothers of children with DD, when compared with those in the POP group (Table 2). A greater proportion of mothers of children in the ASD versus POP group were in the highest tertile of GWG-for-GA *z* scores and GWG rates (Table 2). Based on IOM recommendations, 61.9%, 59.4%, and 59.6% of mothers of children in the ASD, DD, and POP groups, respectively, had an excessive GWG rate, whereas 21.5%, 25.4%, and 21.5% of mothers of children in the ASD, DD, and POP groups, respectively, had inadequate GWG rates (Table 2).

In unadjusted analyses of prepregnancy BMI, maternal overweight, obesity Class 1 and obesity Class 2/3 were associated with higher odds of having a child with ASD or DD (Table 3). Adjustment attenuated these associations (Table 3). However, obesity Class 2/3 remained associated with 87% higher odds of ASD and 61% higher odds of DD in children (Table 3). Obesity Class 2/3 showed a stronger association for ASD without ID than for ASD with ID (Table 3).

In unadjusted GWG-for-GA *z* score models, weight gain in the lowest tertile was associated with higher odds of having a child with DD, whereas weight gain in the highest tertile was associated with higher odds of having a child with ASD, when compared with the middle tertile (Table 4). A similar pattern was seen for ASD with or without ID. After adjustment, these associations were attenuated. Still, weight gain in the highest tertile was associated with 22% higher odds of ASD (Table 4). Both inadequate and excessive rate of GWG per IOM guidelines were associated with higher odds of DD in children but only in unadjusted models (Table 4). Unadjusted and adjusted analyses for GWG rate tertiles showed similar patterns to those observed for GWG-for-GA *z* score tertiles (Supporting Information Table S2). Further adjustment for source of data in GWG models resulted in similar effect estimates (data not shown).

**FIGURE 1:**
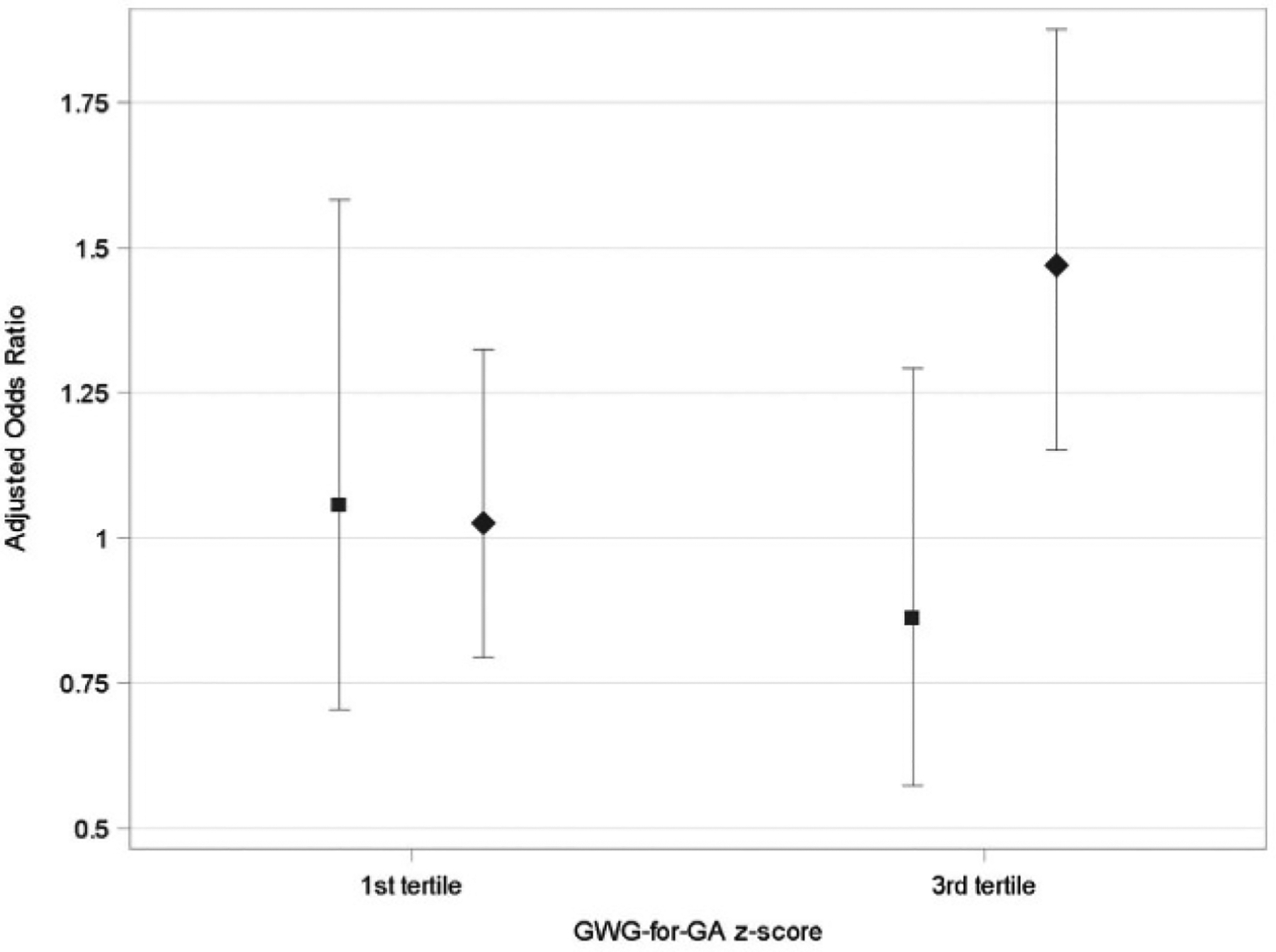
AOR and 95% CI for autism spectrum disorder and GWG-for-GA *z* score tertiles, by child’s sex. Females: square (AOR) and bar (95% CI). Males: diamond (AOR) and bar (95% CI). Reference group is second tertile, which corresponds to a total GWG (for a 40-week pregnancy) between 13.6 kg and 17.2 kg, for a woman with normal prepregnancy BMI. Corresponding values for other BMI categories are listed in Supporting Information Table S1. AOR, adjusted odds ratio; GA, gestational age; GWG, gestational weight gain

No effect modification by child sex was observed for maternal BMI models (data not shown). However, differences by sex were observed for the association between GWG-for-GA *z* score tertiles and ASD (*P* for interaction term = 0.027). Increased odds of ASD were observed for the highest GWG tertile among male children (AOR = 1.47, 95% CI: 1.15–1.88) but not among female children (AOR = 0.86, 95% CI: 0.57–1.29) (Figure 1). A similar pattern was seen for GWG rate (pounds/week) tertiles with increased odds of ASD observed for the highest tertile for male (AOR = 1.32. 95% CI: 1.03–1.67) but not female children (AOR = 0.94, 95% CI: 0.63–1.41; *P* for interaction term = 0.038). Child sex did not modify the association between GWG rate per IOM recommendations and ASD or DD (data not shown). BMI (high vs. normal) did not modify any of the ASD (or DD)-GWG associations (data not shown).

In sensitivity analyses (Supporting Information Table S3) in which children with any indication of ASD were excluded from the DD group, effect estimates for associations between DD and both BMI and GWG variables were similar to those observed for the overall DD group.

## DISCUSSION

Severe maternal obesity (obesity Class 2/3) was associated with nearly double the odds of ASD (with or without ID) and with increased odds of DD, in this large multisite case-control study. Additionally, GWG standardized for gestational age was associated with ASD, particularly in male children; however, no association with DD was observed. When GWG rate was categorized per adherence with clinical recommendations, no associations with either ASD or DD were observed.

Previously, we reported a marginally significant association between prepregnancy obesity, defined as BMI ≥ 30 kg/m^2^, and ASD (22), based on data from the first phase of this study (SEED 1). Here, using a larger sample size, we were able to further subdivide obesity and report that only more severe maternal obesity (Class 2/3) was significantly associated with ASD and DD. Although the association between maternal obesity and ASD (7–9) and DD (8) has been supported in recent meta-analyses, to our knowledge, there was only one previous study that explored categories of obesity severity in relation to ASD (10). That study, conducted in a Danish cohort, reported similar effect estimates for nonsevere (AOR = 1.39, 95% CI: 1.11–1.75) and severe maternal obesity (AOR = 1.38, 95% CI: 0.97–1.97) (10), unlike our study. However, in the Danish cohort study, the proportion of women in the severe obesity category was much smaller (2.3%, vs. 9.8% in SEED), and the proportion of women in the underweight category was higher (4.5%, vs. 3.2% in SEED). Maternal underweight was also associated with ASD in the Danish cohort (10), in contrast to our findings of no association for underweight BMI. Our findings do not support an association between either ASD or DD and maternal prepregnancy BMI in the overweight range, which provides insight into previous studies reporting associations between ASD and a combined maternal overweight/obesity BMI category (7,8,22). Our findings suggest severe obesity (BMI > 35 kg/m^2^) may play a large role in those previous reports. Further examination of ASD and distinct obesity classes is needed in other large cohorts.

Obesity is associated with systemic inflammation, as reflected in elevated cytokine production due to an increase in adipose tissue (40). Inflammatory mediators can cross the blood-placenta barrier and in that way affect fetal neurodevelopment (38,40). Thus, the key mechanisms by which maternal obesity might affect child neurodevelopment relate to maternal inflammation and include neuroinflammation; increased oxidative stress; dysregulated insulin, glucose, and leptin signaling; dysregulated serotonergic and dopaminergic signaling; and perturbations in synaptic plasticity (41). Specifically, dysregulation of placental serotonin production, caused by maternal inflammation, alters neurogenesis and axonal growth in the fetus forebrain, potentially altering the trajectory of fetal brain development (12). Moreover, epigenetic regulation of inflammatory pathways could also be linked to brain changes as a result of perinatal environment (40). Patterns consistent with neurodegeneration, decreased survival of sensory neurons, and decreased neurogenesis were identified in umbilical cord gene expression profiles in fetuses of women with obesity compared with lean women (42).

The prior evidence on associations between GWG and ASD (14–16,22) or DD (22) is limited. Additionally, GWG was characterized in different ways in previous studies, none of which systematically accounted for gestational age, as the current analysis does. Given shortened gestation both reduces the opportunity for maternal weight gain and is associated with poorer neurodevelopment in children (25), it is important to use a GWG metric that accounts for length of gestation (rather than simply limiting to term births). Calculating GWG *z* scores based on gestational age is a novel approach, but its use in perinatal research is increasing and has been proposed as best practice for studying GWG as an exposure (24).

Direct comparison with our previous results on GWG from SEED 1 is not possible because of the different approaches used to characterize GWG and other methodological differences (e.g., primary source of exposure data). Previously, we found that children born at term whose mothers’ total GWG was in the higher quintiles (vs. third quintile) had higher odds of ASD but not of DD (22); the findings were strongest among women who had high prepregnancy BMI.

Although less studied than obesity, excessive GWG has also been associated with higher concentration of inflammatory factors (43). Any potential difference in the mechanisms of obesity and GWG may relate to timing of exposure during fetal life. Children of women with prepregnancy obesity would have been exposed to a heightened inflammatory environment earlier in development or longer than those of women who accumulated excessive weight gain by the end of pregnancy. Interestingly, in this analysis, prepregnancy maternal obesity did not intensify any potential effects of high GWG on ASD, as we previously reported (22), possibly because the current GWG measures took into account maternal prepregnancy BMI.

Consistent with our previous findings, child sex modified the association between high GWG and ASD. Sex differences in the immune responsiveness of the developing brain and placenta offer a potential mechanism that might account for this finding. Specifically, females’ placentae undergo multiple gene expression adaptations, causing a small reduction in growth but enhancing the fetal immune response to the maternal immune challenge, whereas the male placentae response to maternal inflammation involves few changes in gene expression to prioritize ongoing growth (44). This reduced adaptation may make male fetuses more vulnerable to a hostile inflammatory intrauterine environment and may partially explain the higher rate of ASD among males than females.

Caution in the interpretation of our findings is advised, as this study had some limitations, the main one being the lack of medical records data for all participants. Because some of our exposure data came from maternal interviews conducted 3 to 5 years postnatally, (under)reporting and/or recall bias or misclassification could have been introduced. However, our comparison between data among women with both sources revealed excellent agreement for BMI and moderate agreement for GWG. Still, we addressed this limitation by further controlling for source of exposure data in the analyses of GWG and testing whether the source of data modified any observed association. No indication of confounding or effect modification by source of data was found. Second, although final case classification in this study was based on an algorithm with acceptable sensitivity and specificity, the possibility of case misclassification cannot be completely ruled out. Third, although the SEED data allowed adjustment for several relevant confounders, there were important differences across groups in demographic variables (e.g., income, education), which may be associated with unmeasured factors (e.g., poor diet, stress, and environmental toxins); thus, residual confounding may still remain. Although we had limited data for some maternal complications, such as hypertension, we did not include these as potential confounders in our models because they may be in the causal pathway between obesity and/or GWG and ASD. The retrospective nature of our data, coupled with incomplete medical history data, precluded us from conducting a detailed causal path analysis. However, in our previous assessment (22), we ruled out hypertension as a confounder for the associations between both obesity and GWG and ASD or DD. Also, we cannot entirely rule out the potential for selection bias given we did not have sufficient data to classify exposure for 27% of SEED participants. Additionally, many families targeted from the multiple recruitment sources could not be located or contacted. However, assessment of the data from one SEED site with the complete data available to assess nonresponse indicated that many of these families were most likely ineligible for inclusion because they no longer resided in the study catchment area or could not communicate well in English (four of six sites) (45). Additionally, that study found that although nonresponse was associated with younger maternal age, lower maternal education, and non-white race, it was not associated with perinatal factors, such as parity or preterm birth (45). In all analyses, we controlled for these three aforementioned sociodemographic factors.

This study also has important strengths, compared with previous studies. First, a comprehensive developmental in-person assessment using standardized instruments and a validated algorithm were used to define case status, likely reducing case misclassification. Second, this study was conducted in a large, racially and geographically diverse sample. Third, a number of sources of data on covariates and exposure variables created a rich data set. Probably the most important strength of this study is the inclusion of additional components not previously examined, including further subdividing the obesity class by severity and using an innovative GWG metric to account for gestational age and prepregnancy BMI.

Our findings of associations between severe prepregnancy obesity and both ASD and DD and an association between high GWG and ASD among male children indicate the need for future research, including pregnancy inflammation biomarkers and genetic information, to evaluate mechanistic interpretation and identify potential gene-environment interactions. Because maternal BMI and GWG are modifiable factors and generally available in clinical settings, these findings could assist prevention efforts or early intervention for mother-child dyads at high risk.

## Supplementary Material

Tables 1-3

## Figures and Tables

**TABLE 1:** Characteristics of the study sample by offspring case status

	ASD (*n* = 1,159)	DD (*n* = 1,617)	POP (*n* = 1,633)	ASD vs. POP^a^	DD vs. POP^a^
Site				0.4289	0.0704
California	200 (17.3%)	264 (16.3%)	296 (18.1%)		
Colorado	226 (19.5%)	287 (17.7%)	284 (17.4%)		
Georgia	221 (19.1%)	318 (19.7%)	284 (17.4%)		
Maryland	162 (14.0%)	184 (11.4%)	227 (13.9%)		
North Carolina	192 (16.6%)	330 (20.4%)	297 (18.2%)		
Pennsylvania	158 (13.6%)	234 (14.5%)	245 (15.0%)		
Sex of the child				<0.0001	<0.0001
Male	948 (81.8%)	1,059 (65.5%)	833 (51.0%)		
Female	211 (18.2%)	558 (34.5%)	800 (49.0%)		
GA categories				0.0002	<0.0001
Extremely/very preterm (GA <32 weeks)	29 (2.5%)	102 (6.4%)	16 (1.0%)		
Moderate/late preterm (GA 32 to 36 weeks)	105 (9.2%)	192 (12.0%)	102 (6.3%)		
Early/full/late term (GA 37 to 41 weeks)	993 (86.6%)	1,294 (80.6%)	1,484 (91.5%)		
Post-term (GA > 41 weeks)	20 (1.7%)	18 (1.1%)	20 (1.2%)		
Parity (previous live births)				0.0971	0.0001
0	556 (48.1%)	646 (40.2%)	715 (43.9%)		
1	388 (33.5%)	538 (33.5%)	590 (36.2%)		
2+	213 (18.4%)	422 (26.3%)	323 (19.8%)		
Maternal age, y				0.2327	0.0128
<20	25 (2.2%)	58 (3.6%)	51 (3.1%)		
20 to 25	117 (10.1%)	196 (12.1%)	142 (8.7%)		
26 to 29	289 (24.9%)	366 (22.6%)	374 (22.9%)		
30 to 34	413 (35.6%)	539 (33.3%)	603 (36.9%)		
35+	315 (27.2%)	458 (28.3%)	463 (28.4%)		
Maternal race/ethnicity				<0.0001	<0.0001
Non-Hispanic White	572 (49.9%)	879 (54.7%)	1,096 (67.5%)		
Non-Hispanic Black	263 (22.9%)	351 (21.8%)	230 (14.2%)		
Non-Hispanic Asian/multirace/other	143 (12.5%)	130 (8.1%)	149 (9.2%)		
Hispanic	168 (14.7%)	247 (15.4%)	148 (9.1%)		
Maternal education				<0.0001	<0.0001
High school or less	167 (14.4%)	294 (18.2%)	162 (9.9%)		
Some college	375 (32.4%)	474 (29.3%)	359 (22.0%)		
College degree	375 (32.4%)	468 (28.9%)	585 (35.8%)		
Master’s degree or higher	240 (20.7%)	381 (23.6%)	526 (32.2%)		
Household income				<0.0001	<0.0001
Less than 30,000 USD	277 (24.4%)	435 (27.8%)	268 (16.7%)		
30,000 to 70,000 USD	374 (33.0%)	475 (30.4%)	428 (26.6%)		
70,000 to 110,000 USD	267 (23.5%)	372 (23.8%)	488 (30.3%)		
More than 110,000 USD	217 (19.1%)	282 (18.0%)	425 (26.4%)		
Maternal smoking				<0.0001	<0.0001
No smoking during pregnancy	968 (83.5%)	1,380 (85.4%)	1,464 (89.7%)		
Periconceptional smoking	119 (10.3%)	119 (7.4%)	114 (7.0%)		
Smoking late in/throughout pregnancy	72 (6.2%)	117 (7.2%)	54 (3.3%)		

Abbreviations: ASD, autism spectrum disorder; DD, developmental disorders; GA, gestational age; POP, population-based control group.

a χ^2^ test.

**TABLE 2:** Maternal (exposure) weight variables by offspring case status

	ASD (*n* = 1,159)	DD (*n* = 1,617)	POP (*n* = 1,633)	ASD vs. POP	DD vs. POP
Prepregnancy BMI (kg/m^2^)				<0.001^a^	<0.001^a^
Mean (SD)	26.9 (7.4)	26.4 (6.5)	25.2 (5.8)		
Prepregnancy BMI (kg/m^2^)				<0.001^b^	<0.001^b^
Underweight (BMI < 18.5)	38 (3.3%)	48 (3.0%)	53 (3.2%)		
Normal weight (BMI 18.5–24.9)	548 (47.3%)	799 (49.4%)	943 (57.7%)		
Overweight (BMI 25–29.9)	282 (24.3%)	400 (24.7%)	378 (23.1%)		
Obesity Class 1 (BMI 30–34.9)	136 (11.7%)	195 (12.1%)	155 (9.5%)		
Obesity Class 2/3 (BMI 35+)	155 (13.4%)	175 (10.8%)	104 (6.4%)		
GWG-for-GA *z* score				0.005^a^	0.281^a^
Mean (SD)	−0.0 (1.0)	−0.2 (1.1)	−0.1 (1.0)		
GWG-for-GA *z* score^c^				0.017^b^	0.092^b^
1st tertile	331 (29.8%)	543 (34.8%)	508 (32.3%)		
2nd tertile	333 (30.0%)	460 (29.5%)	519 (33.0%)		
3rd tertile	445 (40.1%)	557 (35.7%)	546 (34.7%)		
GWG rate (pounds/week)				0.394^d^	0.028^d^
Median (IQR)	1.1 (0.8, 1.5)	1.0 (0.7, 1.5)	1.1 (0.8, 1.4)		
GWG rate per IOM guidelines				0.401^b^	0.021^b^
Inadequate	243 (21.5%)	401 (25.4%)	353 (22.1%)		
Adequate	187 (16.6%)	242 (15.3%)	292 (18.3%)		
Excessive	699 (61.9%)	939 (59.4%)	951 (59.6%)		
GWG rate (pounds/week)^e^				0.018^b^	0.045^b^
1st tertile	331 (29.3%)	553 (35.0%)	518 (32.5%)		
2nd tertile	356 (31.5%)	468 (29.6%)	537 (33.6%)		
3rd tertile	442 (39.1%)	561 (35.5%)	541 (33.9%)		

Abbreviations: ASD, autism spectrum disorder; DD, developmental disorders; GA, gestational age; GWG, gestational weight gain; IOM, Institute of Medicine; IQR, interquartile range; POP, population-based control group.

a ANOVA *F* test.

b χ^2^ test.

c Second tertile corresponds to total GWG (for a 40-week pregnancy) between 13.6 kg and 17.2 kg for a woman with normal prepregnancy BMI. Corresponding values for other BMI categories listed in Supporting Information Table S1.

d Wilcoxon Mann-Whitney *U* test.

e Second tertile corresponds to GWG rate between 0.91 and 1.26 pounds/week during the second and third trimesters for a woman with normal prepregnancy BMI. Corresponding values for other BMI categories listed in Supporting Information Table S1.

**TABLE 3:** Ciude and adjusted OR and 95% CI for ASD or DD^a^ per prepregnancy maternal BMI categories

	ASD		ASD with ID		ASD without ID		DD	
	Crude	Adjusted^b^	Crude	Adjusted^b^	Crude	Adjusted^b^	Crude	Adjusted^b^
Maternal exposure	OR (95% CI)	OR (95% CI)	OR (95% CI)	OR (95% CI)	OR (95% CI)	OR (95% CI)	OR (95% CI)	OR (95% CI)
	N° cases = 1,118		N° cases = 686		N° cases = 418		N° cases = 1,544	
	N° controls = 1,592		N° controls = 1,592		N° controls = 1,592		N° controls = 1,592	
Prepregnancy BMI								
Underweight	1.09 (0.70–1.71)	0.99 (0.62–1.58)	1.03 (0.59–1.79)	0.89 (0.50–1.60)	1.04 (0.56–1.94)	1.04 (0.55–1.96)	0.98 (0.64–1.48)	0.89 (0.58–1.37)
Normal weight	Ref	Ref	Ref	Ref	Ref	Ref	Ref	Ref
Overweight	1.28 (1.06–1.54)	1.12 (0.92–1.37)	1.43 (1.15–1.79)	1.17 (0.92–1.48)	1.03 (0.78–1.35)	1.03 (0.78–1.37)	1.26 (1.06–1.49)	1.09 (0.91–1.30)
Obesity Class 1	1.46 (1.13–1.89)	1.15 (0.88–1.52)	1.79 (1.34–2.39)	1.25 (0.92–1.72)	0.98 (0.66–1.45)	0.99 (0.66–1.49)	1.47 (1.16–1.86)	1.21 (0.95–1.55)
Obesity Class 2/3	2.54 (1.93–3.34)	1.87 (1.40–2.51)	2.69 (1.97–3.67)	1.71 (1.22–2.40)	2.34 (1.64–3.35)	2.30 (1.57–3.38)	2.07 (1.59–2.69)	1.61 (1.22–2.13)

Abbreviations: ASD, autism spectrum disorder; DD, developmental disorders; ID. intellectual disability (IQ ≤ 70); OR, odds ratio.

a Compared with population-based control group (*n* = 1,633).

b Adjusted for maternal age, education, race/ethnicity, parity, smoking, income, and site (categorized as in Table 1).

**TABLE 4:** Crude and adjusted OR and 95% CI for ASD or DD^a^ per gestational weight gain categories

	ASD		ASD with ID		ASD without ID		DD	
	Crude	Adjusted^b^	Crude	Adjusted^b^	Crude	Adjusted^b^	Crude	Adjusted^b^
Maternal exposure	OR (95% CI)	OR (95% CI)	OR (95% CI)	OR (95% CI)	OR (95% CI)	OR (95% CI)	OR (95% CI)	OR (95% CI)
	N° cases = 1,069		N° cases = 650		N° cases = 407		N° cases = 1,492	
	N° controls = 1,533		N° controls = 1,533		N° controls = 1,533		N° controls = 1,533	
GWG-for-GA *z* score^c^								
1st tertile	1.03 (0.85–1.26)	0.97 (0.79–1.19)	1.03 (0.81–1.30)	0.92 (0.72–1.18)	1.03 (0.77–1.36)	1.00 (0.75–1.34)	1.22 (1.02–1.45)	1.10 (0.91–1.32)
2nd tertile	Ref	Ref	Ref	Ref	Ref	Ref	Ref	Ref
3rd tertile	1.29 (1.07–1.56)	1.22 (1.00–1.49)	1.24 (0.99–1.55)	1.17 (0.92–1.48)	1.35 (1.03–1.75)	1.26 (0.96–1.65)	1.16 (0.97–1.38)	1.14 (0.95–1.36)
	N° cases = 1,089		N° cases = 666		N° cases = 411		N° cases = 1,512	
	N° controls = 1,556		N° controls = 1,556		N° controls = 1,556		N° controls = 1,556	
GWG rate per IOM guidelines								
Inadequate	1.08 (0.84–1.39)	0.97 (0.75–1.26)	1.23 (0.91–1.66)	1.01 (0.73–1.39)	0.89 (0.63–1.26)	0.89 (0.62–1.26)	1.41 (1.12–1.77)	1.19 (0.94–1.50)
Adequate	Ref	Ref	Ref	Ref	Ref	Ref	Ref	Ref
Excessive	1.14 (0.92–1.41)	1.08 (0.87–1.34)	1.28 (0.99–1.66)	1.17 (0.89–1.53)	0.96 (0.72–1.28)	0.92 (0.68–1.23)	1.23 (1.01–1.50)	1.18 (0.97–1.45)

ASD, autism spectrum disorder; DD, developmental disorders; GA, gestational age; GWG, gestational weight gain; ID, intellectual disability (IQ ≤ 70); IOM, Institute of Medicine; OR, odds ratio.

a Compared with population-based control group (*n* = 1,573 for GWG-for-GA *z* score; *n* = 1,633 for GWG rate per IOM recommendations).

b Adjusted for maternal age, education, race/ethnicity, parity, smoking, income, and site (categorized as in Table 1).

c Second tertile corresponds tototal GWG (for a 40-week pregnancy) between 13.6 kg and 17.2 kg, for a woman with normal prepregnancy BMI. Corresponding values for other BMI categories listed in Supporting Information Table S1.

## Study Importance

### What is already known?

- Maternal prepregnancy obesity has been linked to risk of autism spectrum disorder in offspring.

### What does this study add?

- Severe maternal prepregnancy obesity is specifically associated with autism spectrum disorder and other developmental disorders in children.
- Maternal gestational weight gain, when accounting for duration of pregnancy, is associated with autism spectrum disorder, particularly in male children.

### How might these results change the direction of research or the focus of clinical practice?

- Maternal BMI and gestational weight gain are potentially modifiable factors routinely measured and monitored in clinical settings. Therefore, these findings could be helpful for targeting and implementing early intervention for mother-child dyads at high risk.
