# Supplementary material for: Maternal prepregnancy weight and gestational weight gain in association with autism and developmental disorders in offspring
Source: Obesity (Silver Spring). Author manuscript; Available in PMC 2022 Sep 1. (PMC9186321; doi:10.1002/oby.23228)
Supplement: Tables 1-3 [file NIHMS1802752-supplement-Tables_1-3.docx]

Table S1. BMI-specific cut-offs and corresponding GWG values to create GWG categorical (tertiles) variables

| GWG-for-GA z-score | Percentile 33th^1^ | Corresponding GWG (kg)^2^ | Percentile 66th^1^ | Corresponding GWG (kg)^2^ |
| --- | --- | --- | --- | --- |
| Underweight | -0.45700 | 12.9 | 0.45402 | 17.8 |
| Normal weight | -0.50775 | 13.6 | 0.14035 | 17.2 |
| Overweight | -0.57011 | 11.7 | 0.13768 | 16.9 |
| Obesity Class 1 | -0.23951 | 11.0 | 0.39751 | 16.3 |
| Obesity Class 2 | -0.21210 | 8.0 | 0.32171 | 13.1 |
| Obesity Class 3 | -0.47705 | 2.0 | 0.29973 | 10.4 |
| GWG rate (lb/week)^3^ | Percentile 33th^1^ |  | Percentile 66th^1^ |  |
| Underweight | 0.91 |  | 1.32 |  |
| Normal weight | 0.91 |  | 1.26 |  |
| Overweight | 0.79 |  | 1.22 |  |
| Obesity Class 1 | 0.74 |  | 1.18 |  |
| Obesity Class 2 | 0.44 |  | 0.91 |  |
| Obesity Class 3 | -0.02 |  | 0.69 |  |

^1^Among mothers of children in the general population (POP) group, in that specific BMI category

^2^For a 40-week pregnancy

^3^Pounds per week during the 2^nd^ and 3^rd^ trimesters

Table S2. Crude and adjusted odds ratios (ORs) and 95% confidence intervals (CI) for ASD or DD^1^ per maternal gestational weight gain rate (pounds/week) tertiles^1^

| Maternal exposure | ASD | | ASD with ID | | ASD without ID | | DD | |
| --- | --- | --- | --- | --- | --- | --- | --- | --- |
|  | Crude | Adjusted^2^ | Crude | Adjusted^2^ | Crude | Adjusted^2^ | Crude | Adjusted^2^ |
|  | OR (95% CI) | OR (95% CI) | OR (95% CI) | OR (95% CI) | OR (95% CI) | OR (95% CI) | OR (95% CI) | OR (95% CI) |
|  | N° cases=1089  N° controls=1556 | | N° cases=666  N° controls=1556 | | N° cases=411  N° controls=1556 | | N° cases=1512  N° controls=1556 | |
| GWG rate^3^ |  |  |  |  |  |  |  |  |
| 1^st^ tertile | 0.96 (0.79-1.17) | 0.89 (0.73-1.09) | 1.02 (0.81-1.28) | 0.91 (0.71-1.17) | 0.86 (0.65-1.13) | 0.84 (0.63-1.12) | 1.20 (1.01-1.43) | 1.06 (0.88-1.27) |
| 2^nd^ tertile | Ref | Ref | Ref | Ref | Ref | Ref | Ref | Ref |
| 3^rd^ tertile | 1.23 (1.02-1.48) | 1.14 (0.94-1.39) | 1.24 (0.99-1.54) | 1.14 (0.90-1.44) | 1.18 (0.91-1.53) | 1.10 (0.84-1.44) | 1.18 (0.99-1.40) | 1.13 (0.94-1.35) |

ASD: Autism Spectrum Disorder; DD: Developmental disorders; GWG: Gestational weight gain; ID: Intellectual disability (IQ≤70).

^1^Compared to the population-based control group (POP; n=1556).

^2^Adjusted for maternal age, education, race/ethnicity, parity, smoking, income, and site (categorized as in Table 1).

^3^Second tertile corresponds to 0.91 to 1.26 lb/week during the 2^nd^ and 3^rd^ trimesters, for a woman with normal pre-pregnancy weight.

Table S3. Crude and adjusted odds ratios (ORs) and 95% confidence intervals (CI) for DD subgroup without previous ASD diagnosis nor ASD-like characteristics

| Maternal exposure | Crude  OR (95% CI) | Adjusted^1^  OR (95% CI) |
| --- | --- | --- |
|  | N° cases=1267 / N° controls=1592 | |
| Pre-pregnancy BMI category |  |  |
| Underweight | 1.01 (0.66, 1.57) | 0.96 (0.61, 1.49) |
| Overweight | 1.18 (0.98, 1.41) | 1.02 (0.84, 1.23) |
| Normal weight | Ref | Ref |
| Obesity class 1 | 1.40 (1.09, 1.79) | 1.16 (0.90, 1.50) |
| Obesity class 2 & 3 | 1.86 (1.41, 2.46) | 1.47 (1.10, 1.96) |
|  |  |  |
|  | N° cases=1227 / N° controls=1533 | |
| GWG-for-GA Z-score^2^ |  |  |
| 1^st^ tertile | 1.24 (1.03, 1.49) | 1.13 (0.93, 1.37) |
| 2^nd^ tertile | Ref | Ref |
| 3^rd^ tertile | 1.13 (0.94, 1.36) | 1.13 (0.94, 1.37) |
|  |  |  |
|  | N° cases=1241 / N° controls=1556 | |
| GWG rate per IOM recommendations |  |  |
| Inadequate | 1.35 (1.06, 1.71) | 1.15 (0.90, 1.47) |
| Adequate | Ref | Ref |
| Excessive | 1.12 (0.91, 1.38) | 1.09 (0.88, 1.34) |
|  |  |  |
|  | N° cases=1241 / N° controls=1556 | |
| GWG rate (pounds/week)^3^ |  |  |
| 1^st^ tertile | 1.23 (1.02, 1.48) | 1.10 (0.91, 1.34) |
| 2^nd^ tertile | Ref | Ref |
| 3^rd^ tertile | 1.17 (0.98, 1.41) | 1.15 (0.95, 1.39) |
|  |  |  |

ASD: Autism Spectrum Disorder; BMI: body mass index; DD: Developmental disorders; GA: Gestational age; GWG: Gestational weight gain; IOM: Institute of Medicine.

^1^Adjusted for maternal age, education, race/ethnicity, parity, smoking, income and site (categorized as in Table 1).

^2^Second tertile corresponds to total GWG (for a 40-week pregnancy) between 13.6 kg and 17.2 kg, for a woman with normal pre-pregnancy weight.

^3^Second tertile corresponds to GWG rate between 0.91 and 1.26 lb/week during the 2^nd^ and 3^rd^ trimesters, for a woman with normal pre-pregnancy weight.
